# Interpreting text messages with graphic facial expression by deaf and hearing people

**DOI:** 10.3389/fpsyg.2015.00383

**Published:** 2015-04-02

**Authors:** Chihiro Saegusa, Miki Namatame, Katsumi Watanabe

**Affiliations:** ^1^Research Center for Advanced Science and Technology, The University of Tokyo, Tokyo, Japan; ^2^Department of Synthetic Design, Tsukuba University of Technology, Tsukuba, Japan

**Keywords:** smileys, text interpretation, chat, social signals, earnestness, deaf, hearing

## Abstract

In interpreting verbal messages, humans use not only verbal information but also non-verbal signals such as facial expression. For example, when a person says “yes” with a troubled face, what he or she really means appears ambiguous. In the present study, we examined how deaf and hearing people differ in perceiving real meanings in texts accompanied by representations of facial expression. Deaf and hearing participants were asked to imagine that the face presented on the computer monitor was asked a question from another person (e.g., do you like her?). They observed either a realistic or a schematic face with a different magnitude of positive or negative expression on a computer monitor. A balloon that contained either a positive or negative text response to the question appeared at the same time as the face. Then, participants rated how much the individual on the monitor really meant it (i.e., perceived earnestness), using a 7-point scale. Results showed that the facial expression significantly modulated the perceived earnestness. The influence of positive expression on negative text responses was relatively weaker than that of negative expression on positive responses (i.e., “no” tended to mean “no” irrespective of facial expression) for both participant groups. However, this asymmetrical effect was stronger in the hearing group. These results suggest that the contribution of facial expression in perceiving real meanings from text messages is qualitatively similar but quantitatively different between deaf and hearing people.

## Introduction

Interpreting verbal messages, perceiving others’ real meaning, and responding to them appropriately are important in successful communication. In some cases, the meanings are communicated directly in a verbal form, but in most cases, we infer them by cues that are provided explicitly or implicitly (Duncan, 1969). Most of the cues that signal the real meanings might be in visual or auditory modalities. For instance, expressions of emotion in the face and through body movement would be cues in the visual modality, whereas prosody such as speed, intonation, and accent of the voice would be cues in the auditory modality (Scherer et al., 1991; Banse and Scherer, 1996).

The recent increase of human–computer interaction and human–human communication via computer requires a person to use similar yet slightly different communication styles than a face-to-face communication. The major difference is the amount of information and relative contribution of different sensory modalities that are being accessed. For example, in a computer-mediated communication such as e-mails and online chats, we convey our thoughts mainly with text messages. Thus, there are fewer non-verbal cues for emotion that would otherwise play an important role in inferring real meaning in face-to-face communications. Emoticons and avatars are used to replace non-verbal cues in computer-mediated communications. It has been reported that emotional expression by such methods can modify inference of meanings from text messages (Derks et al., 2008).

Indeed, facial expression is a rich source of information on emotional states for the beholder and is considered the most important cue. It has been proposed that the perception of human facial expression is universal regardless of culture in most cases (Ekman et al., 1969; Ekman and Friesen, 1971), with some cognitive and behavioral differences in interpreting facial expressions, for example, with regard to perceiving the intensity of emotions (Ekman et al., 1987), integrating social context into emotion judgment (Masuda et al., 2008), mental representations (Jack et al., 2012), and fixation maps (Jack et al., 2009). Since there are many cultural differences in the cognitive process in addition to the difference in cognition of facial expression of emotion (for review, Nisbett and Masuda, 2003), the findings may reflect a general difference in cognitive process across cultures rather than differences specific to facial expression. Facial expression is useful not only for perceiving emotional states of the communicator but also in judging deception (Feldman et al., 1979). For instance, Ekman and Friesen (1974) demonstrated that people utilized both facial and body cues when detecting deception from videotaped interviews in which models acted out both honest and deceptive responses.

Similar to the cross-cultural commonalities and differences, deaf people and people with normal hearing share a common perception of expression of emotion, while using different eye movement paths in collecting information from the face (Watanabe et al., 2011). In addition, previous research has demonstrated differences between deaf people and hearing people in the perceptual and cognitive processing of faces when memorizing (Arnold and Murray, 1998) and discriminating faces, especially in discriminating the local features of faces (Bettger et al., 1997; McCullough and Emmorey, 1997). As McCullough et al. (2005) discussed, such differences might be due to deaf people’s constant attention to componential facial features versus hearing people’s constant attention to holistic facial information, and these differences might influence and/or be influenced by other cognitive processes, for example, how the facial expression information is integrated with information from other modalities.

Although facial expression is essential to understanding the emotional state of others, it is rarely used independently. Rather, it is integrated with other information. For example, in the perception of real intention based on verbal, vocal, and visual input, the perception of positivity in the affective message expressed in one modality is discounted when there is contradictory input from other modalities (Bugental et al., 1970; Friedman, 1979). However, the difference in the cognitive processing of facial expression between deaf and normal hearing people may result in a different usage of facial expression information when integrating it with information from other modalities to infer real meanings from text messages made by others.

Thus in the current study, we aimed to improve understanding of how the use of facial expression in perceiving real meanings from text messages differs between deaf and hearing adults, depending on combinations of verbal information presented as texts together with facial expressions of emotion to convey either consistent or contradictory contents.

We had two hypotheses for the current study. The first refers to the communication strategy in deaf people. In addition to the difference in gaze strategy during processing emotional expression of the face (Watanabe et al., 2011), there are a few reports suggesting a difference between deaf and hearing people in the usage of non-verbal cues when communicating with others (Barnett, 2002). For example, it was reported that differences in interpreting non-verbal gestures including body posture and facial expression may lead to misunderstandings between a deaf patient and his or her hearing physician (Barnett, 2002). However, to our knowledge, the exact contexts and situations for such misunderstandings remain unclear. In the current study, we investigated how facial emotion expression on a computer monitor would affect the inference of real meaning behind the explicitly presented text responses. Our prediction was that deaf people regard visual facial expressions as more useful sources for interpreting the text messages because they have less access to auditory cues (e.g., prosodic sounds). The second hypothesis refers to the politeness assumption (Brown and Levinson, 1987); that is, how a participant assumes the person/agent in the conversation as being polite may depend on the conversation context. The communication strategy might differ depending on the situation, especially when the response is a negative one. In order to examine this, we chose the following two questions: Asking someone for a favor and asking about liking another person. Asking someone for a favor occurs in a conversation between two persons. A negative response would not be desirable for the questioner. Such a situation requires the assumption that the answerer would avoid explicitly expressing a negative response but would employ an implicit way (e.g., negative facial expression). On the other hand, asking about liking another person who is not present in the conversation would threaten the relationship between the pair less although it might still not be socially desirable. Therefore, we expected that the influence of emotion expression as a non-verbal cue would be smaller. We further predicted that, if the response was positive, such a difference between emotion expressions would not be observed.

In our experiment, both realistic and schematic faces were investigated because we assumed that, irrespective of hearing ability and history, there might be a general difference in the amount of emotional signals that can be extracted from these types of faces (Wallraven et al., 2007) and a difference in strategy that observers take while seeing them. For example, it was reported that gaze behavior for recognizing schematically drawn faces and natural-looking faces is different, and that schematically drawn faces facilitate analytical processing (Schwarzer et al., 2005). Further evidence for the different strategies can be found in face recognition (Rosset et al., 2010) and in emotional processing of schematic faces in patients with autistic spectrum disorder (Rosset et al., 2008). In addition, understanding the possible differences between face types would be informative when applying these findings to human–computer interaction because the agents on the computer are often abstract representations of a person.

Although a computer-generated (CG) face is not animated and thus may not have an intention as in the pragmatic and philosophical literature (Grice, 1969; Sperber and Wilson, 2002), humans tend to extract meaning from what is displayed on the face (Öhman, 2002). Thus in the current study, we investigated perception of the real meaning of what was conveyed via verbal message and emotion expression of the face with different levels of consistency. We were especially focused on whether participants’ inferences of the meaning that is explicitly (e.g., verbally) explained would be affected by emotional valence that is displayed on the face.

## Materials and Methods

### Participants

The participants included 20 deaf Japanese people and 36 Japanese people with normal hearing function. All deaf participants were undergraduate students at the Tsukuba University of Technology, where hearing loss of 60 dB or more is one of the requirements for admission. Data from five hearing participants were excluded because the session for expression rating was not completed. The remaining data from 20 deaf participants (6 males and 14 females; mean age = 21.1 years old, SD = 1.0) and 31 participants with normal hearing function (20 males and 11 females; mean age = 21.2 years old, SD = 1.6) were used for the analyses.

### Visual Stimuli

**FIGURE 1:**
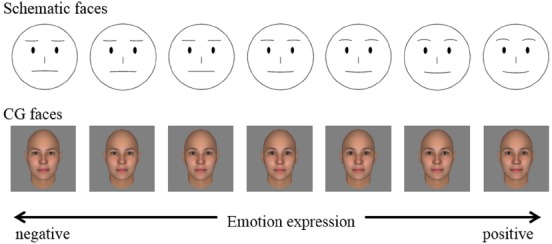
**Facial stimuli used in the experiment**.

Schematic faces and CG faces with a stepwise emotional expression manipulation were used in the experiment (Figure 1). In the schematic faces, to express positive and negative emotions, the shapes and height of the eyebrows and mouth line were manipulated. For positive expressions, the middle points of the eyebrows were placed above the ends of the eyebrows, and the middle point of the mouth line was placed below the ends of the mouth. Conversely, for negative expressions, the middle points of the eyebrows were placed below the ends of the eyebrows, and that of the mouth line was placed above the ends. The heights of the middle points were systematically manipulated and connected with the end points (of eyebrows or mouth line) by using a spline curve. For CG faces, a face generated by the FaceGen Modeler 3.3 (Singular Inversions, Toronto, ON, Canada) with average race and average gender was used as default. Then, the face was morphed by changing to “SmileClosed” to generate positive expressions or changing to “Disgust” to generate negative expressions. “Smile-Closed” and “Disgust” are parameters defined in the FaceGen Modeler.

To determine the optimal range of emotion expression to be used in the experiment, we conducted a preliminary experiment with faces with 11 levels of emotion expression. Thirteen hearing participants inferred the meaning behind the text messages displayed along with the face in an analogous way to the main experiment. For CG faces, negative emotion expressions were created with changing levels of “Disgust” in the FaceGen Modeler. Positive emotion expressions were created with changing levels of “SmileClosed.” Thus, the set of CG faces consisted of 11 faces including 5 negative and 5 positive expressions and one neutral expression. The results of the preliminary experiment indicated that participants’ evaluation drastically changed even with the mild expressions and that for the stronger expressions the evaluations tended to saturate. Thus, based on these results, we chose the range of emotion expressions being used in our main experiment. The range of expressions selected for CG faces consisted of emotion expression magnitudes of 0.13, 0.27, and 0.40 for both “Disgust” and “SmileClosed” within the settings of FaceGen Modeler in addition to the original neutral face. For the schematic faces, levels 5 and 8 used in the preliminary experiment (with 1 the most negative and 11 the most positive emotion expression of the faces that were used) were selected as the minimum and maximum expressions, respectively, and seven levels of emotional expressions were prepared to be distributed evenly within the range.

### Procedure

**FIGURE 2:**
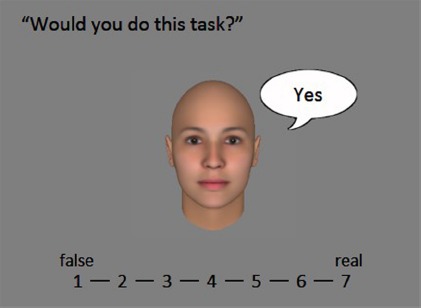
**Sample screen for evaluating earnestness.** The texts were presented in Japanese in the experiment.

The experiment consisted of two blocks. In the first experimental block, for each trial, a schematic or a CG face was presented at the center of the monitor, with a question directed to the face presented at the top and a response to the question in a cartoon balloon. Participants were asked to rate whether the response shown in a cartoon balloon represented the person’s genuine feeling based on a 7-point Likert scale from 1 = false to 7 = real (perceived earnestness; see Figure 2). There were two sets of questions and responses (positive and negative) used in the experiment. All the questions and responses were presented in Japanese. In one set, the question was “Do you like her? (*kanojo no koto suki?* in Japanese),” and a positive response was “Yes (*suki*)” while a negative response was “No (*kirai*)” (negative). In the other set, the question was “Would you do this task? (*kono shigoto yatte kureru?*),” and a positive response was “Yes (*iiyo*)” while a negative response was “No (*iyada*).” The types of face stimuli (schematic or CG) and questions were fixed in sub-blocks in which seven levels of expression of emotion from negative to positive and two types of response (negative/positive) were presented in a randomized order. The order of sub-blocks was counterbalanced among participants. This was followed by the second experimental block, where the same faces as in the first block were presented one by one on the monitor, and participants were asked to judge how positive the emotion expressed on each face was, using a 7-point Likert scale ranging from 1 = negative to 7 = positive. This experimental block consisted of two sub-blocks, in which schematic and CG faces were presented separately. Each individual face was presented twice within a sub-block in randomized order. Experiments were written in Matlab using the Psychophysics Toolbox extensions (Brainard, 1997; Pelli, 1997; Kleiner et al., 2007). The instructions were given in written texts for both groups of participants. Although the specific situations of the contexts were not described in the instruction, most participants reported that they spontaneously took the situation as representing the messages and faces created by a third party in a face-to-face scenario. The procedure was approved by the internal review board of the University of Tokyo.

## Results

### Ratings of Emotion Expressed on the Faces

To check if the emotional expression manipulation was successful, effects of pre-assigned emotion expression level (1: the most negative to 7: the most positive), type of face (schematic/CG), and participants’ hearing status (deaf/hearing) were examined by a three-way repeated measure analysis of variance (ANOVA), conducted on the ratings for positivity/negativity of emotions expressed in the faces.

**FIGURE 3:**
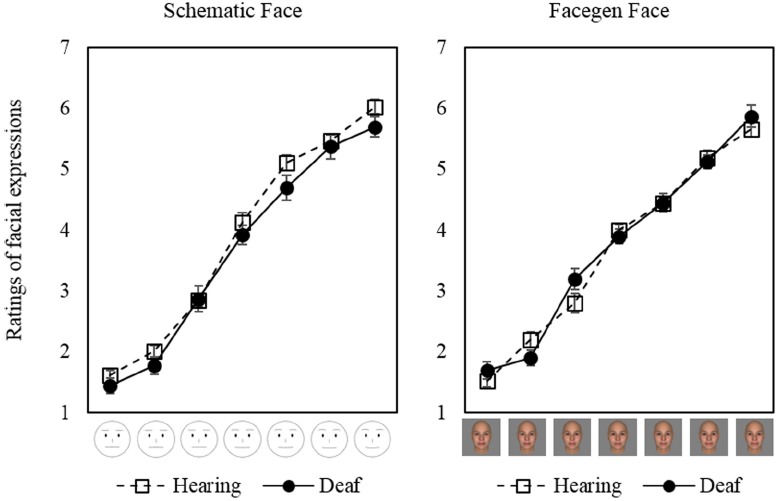
**Ratings of facial expressions.** Error bars represent standard errors of means.

Ratings of perceived positivity/negativity of emotion expressed in the schematic and CG faces increased as the pre-assigned level of expressed emotion increased (Figure 3). This indicated the manipulation of the expression of emotion was successful both in schematic faces and in CG faces. Results of the ANOVA demonstrated that the main effect of pre-assigned level of expressed emotion on the ratings was significant [*F*(3.31, 162.2) = 607.6, *p* < 0.001, ${\text{η}}_{\text{p}}^{\text{2}}$ = 0.93, using Greenhouse–Geisser corrected degrees of freedom], such that faces manipulated to appear more positive were perceived as more positive. A significant interaction effect was found between stimulus type and emotional expression level [*F*(4.69, 229.9) = 4.08, *p* < 0.01, ${\text{η}}_{\text{p}}^{\text{2}}$ = 0.078] while the main effect of stimulus type was not significant [*F*(1, 49) = 0.63, *p* = 0.43, ${\text{η}}_{\text{p}}^{\text{2}}$ = 0.013]. This indicates that the perceived positivity of the expressed emotions might differ between CG and schematic faces. Neither main effect nor interactions associated with participants’ hearing status were significant: *F*(1, 49) = 1.46, *p* = 0.23, ${\text{η}}_{\text{p}}^{\text{2}}$ = 0.029 for the main effect of hearing status, *F*(1, 49) = 1.78, *p* = 0.19, ${\text{η}}_{\text{p}}^{\text{2}}$ = 0.035 for the interaction between stimulus type and hearing status, *F*(3.31, 162.2) = 1.41, *p* = 0.24, ${\text{η}}_{\text{p}}^{\text{2}}$ = 0.028 for the interaction between pre-assigned emotion expression level and hearing status, and *F*(4.69, 229.9) = 1.03, *p* = 0.40, ${\text{η}}_{\text{p}}^{\text{2}}$ = 0.021 for the three-way interaction, using Greenhouse–Geisser corrected degrees of freedom in calculating the latter two *F*-values.

To further examine if there were differences in emotion expression recognition between deaf and hearing participants, we performed a regression analyses within each participant on the ratings of perceived emotion with the pre-assigned emotion expression level a descriptive factor, separately for schematic and CG faces. Then, the coefficients of the pre-assigned level were compared across stimulus type and participant groups with a repeated-measure Bayesian ANOVA with using JASP 0.5 (Love et al., 2014). The results indicated neither significant effects of stimulus type (*BF*_10_ = 0.25; substantial evidence for *H*_0_), participant group (*BF*_10_ = 0.12; substantial evidence for *H*_0_), nor a significant interaction between these two factors (*BF*_10_ = 0.11; substantial evidence for *H*_0_). The results supported that deaf and hearing participants did not differ in interpreting facial emotional expression of the faces used in the experiment.

### Inferring Real Meaning from Text Messages Accompanied with Facial Expression

For perceived earnestness, the ratings for the trials where the facial character responded negatively to the questions (i.e., response was “No”) were inverted before being used in the analyses. Thus, in the ratings after this manipulation, one indicates that participants estimated the response’s real meaning as negative, while seven indicates that participants estimated the response’s real meaning as positive, regardless of congruency between the response shown in the balloon and the estimated real meaning. Then, the influence of the facial expression on the participants’ interpretation of the text response shown in the balloon (positive or negative) and the influence of the participants’ hearing status were examined by a mixed-design ANOVA with stimulus type (schematic or CG), question asked, text response (positive or negative), and pre-assigned level of expressed emotion as within-participant factors and participants’ group (deaf or hearing) as between-participants factor.

**FIGURE 4:**
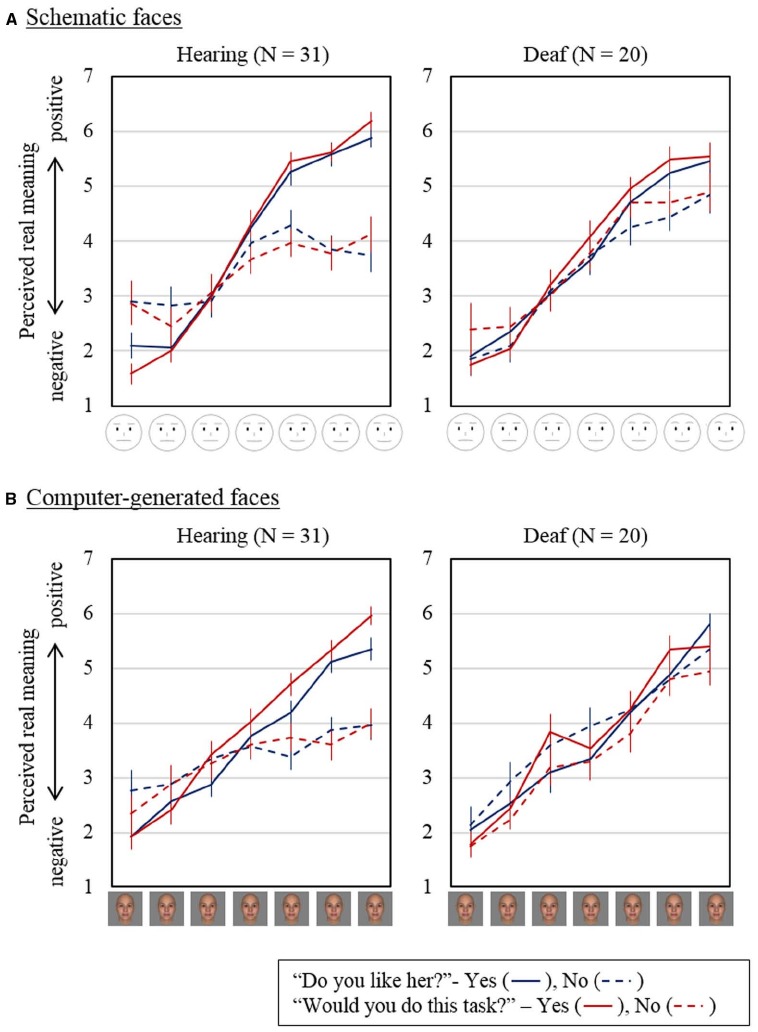
**Perceived real meaning inferred from the combination of text and facial expression for schematic faces (A) and for computer-generated faces (B).** Blue lines are for the question “Do you like her?” with a positive response “Yes” (blue solid lines) and a negative response “No” (blue dashed lines). Red lines are for the question “Would you do this task?” with a positive response “Yes” (red solid lines) and a negative response “No” (red dashed lines). Error bars represent standard errors of the means.

Generally, as shown in Figure 4, the texts with positive facial expression were interpreted as having more positive real meaning, regardless of stimulus type, question, or response presented in the balloon. The ANOVA results that demonstrated the significant main effect of the pre-assigned level of emotion expressed on the face [*F*(2.12, 103.8) = 189.3, *p* < 0.001, ${\text{η}}_{\text{p}}^{\text{2}}$ = 0.79, using Greenhouse–Geisser corrected degrees of freedom] supported this finding. Regarding the effect of response type, the main effect of response type and the interaction between response type and expressed emotion level were both significant [*F*(1, 49) = 5.10, *p* < 0.05, ${\text{η}}_{\text{p}}^{\text{2}}$ = 0.094, for the main effect; *F*(2.30, 112.7) = 18.4, *p* < 0.001, ${\text{η}}_{\text{p}}^{\text{2}}$ = 0.273 for the interaction, using Greenhouse–Geisser corrected degrees of freedom], suggesting that the effect of facial expression differed depending on whether the response was positive or negative. When the response was negative (dashed lines in Figure 4), the ratings tended to be low. This indicates that if the response was negative, the real meaning was judged as negative irrespective of the facial expression. Further, this interaction significantly differed between the participant groups [*F*(2.30, 112.7) = 6.15, *p* < 0.005, ${\text{η}}_{\text{p}}^{\text{2}}$ = 0.112, using Greenhouse–Geisser corrected degrees of freedom]. Thus, this indicates a difference between deaf people and hearing people in how facial expression was integrated into the evaluation of perceived earnestness (and negativity of the text messages). The ANOVA results also demonstrated a significant interaction between face type and expressed emotion level [*F*(6, 294) = 7.55, *p* < 0.001, ${\text{η}}_{\text{p}}^{\text{2}}$ = 0.13], but this might be an artifact from the different interpretation of emotion depending on face type found in the positivity/negativity ratings of the expressed emotion. The interaction between face type, context, and participants was also significant [*F*(1, 49) = 5.06, *p* < 0.05, ${\text{η}}_{\text{p}}^{\text{2}}$ = 0.094]. All remaining main effects and interactions, including the main effect of participants’ hearing status [*F*(1, 49) = 0.067, *p* = 0.80, ${\text{η}}_{\text{p}}^{\text{2}}$ = 0.001], were not significant or only marginally significant.

We also conducted separate ANOVAs for each group to interpret the significant interactions. The main effect of facial expression was significant both in hearing [*F*(1.97, 59.1) = 94.2, *p* < 0.001, ${\text{η}}_{\text{p}}^{\text{2}}$ = 0.76] and deaf participants [*F*(1.97, 37.5) = 102.2, *p* < 0.001, ${\text{η}}_{\text{p}}^{\text{2}}$ = 0.84; both using Greenhouse–Geisser corrected degrees of freedom]. Thus, it was confirmed that the emotion expression had a significant influence on how the text response was interpreted.

The main effect of the response type was significant only in hearing participants [*F*(1, 30) = 6.61, *p* < 0.05, ${\text{η}}_{\text{p}}^{\text{2}}$ = 0.18 for hearing; *F*(1, 19) = 0.63, *p* = 0.44, ${\text{η}}_{\text{p}}^{\text{2}}$ = 0.032 for deaf]. This might reflect that a significant interaction between response type and expression level was found in hearing participants [*F*(2.07, 62.0) = 23.3, *p* < 0.001, ${\text{η}}_{\text{p}}^{\text{2}}$ = 0.44], while the interaction was only marginally significant in deaf participants [*F*(2.67, 50.8) = 2.41, *p* = 0.084, ${\text{η}}_{\text{p}}^{\text{2}}$ = 0.11]. These results may indicate that the rating was differently influenced by emotional expressions depending on the content of verbal response in hearing participants and resulted in the significant main effect of the response type.

Significant interactions between face type and expression level were found in both participant groups [*F*(6, 180) = 5.19, *p* < 0.001, ${\text{η}}_{\text{p}}^{\text{2}}$ = 0.15 for hearing; *F*(6, 114) = 3.26, *p* < 0.01, ${\text{η}}_{\text{p}}^{\text{2}}$ = 0.15 for deaf]. As already discussed, the influence of pre-assigned expression level on perceived positivity/negativity differed between face types. Thus, the interactions between face type and expression level in the rating might reflect the significant interaction in evaluation of facial expression itself rather than the difference in the process of integrating the facial emotion expression to interpret the real meaning.

Only in deaf participants, significant interactions between face type, context, and response type [*F*(1, 19) = 5.03, *p* < 0.05, ${\text{η}}_{\text{p}}^{\text{2}}$ = 0.21] and between context, response type, and emotion expression of the face [*F*(6, 114) = 2.44, *p* < 0.05, ${\text{η}}_{\text{p}}^{\text{2}}$ = 0.11] were found. *Post hoc* comparisons of these interactions indicated that the ratings of schematic faces in the situation where a positive response was given to the question “Do you like her?” were significantly higher than those of CG faces [difference of mean = (–0.39, 95% CI (–0.70, (–0.075), *p* < 0.05], while the ratings for different face types were not significantly different for the question “Would you do this task?” [difference of mean = 0.27, 95% CI (–0.20, 0.73), *p* = 0.231, both with Bonferroni correction]. The ratings with the two most positive emotion expressions were significantly higher when interpreting positive verbal responses than when interpreting negative responses but only in the trials with CG faces [difference of mean = 0.68, 95% CI (0.14, 1.21), *p* < 0.05 for the second-most positive expression; difference of mean = 0.55, 95% CI (0.019, 1.08), *p* < 0.05 for the most positive expression]. These differences were not found when interpreting the verbal responses presented with schematic faces.

### An Ordinal Logistic Regression Model for Predicting Perceived Real Meaning of the Verbal Responses

To investigate possible factors that affected the ratings of positivity of the real meaning, an ordinal logistic regression analysis was performed with all the possible factors (participant group, context, type of face, and type of text response), covariate (emotion expression level of face), as well as all possible interactions between them. Then, we restructured the model by using the factors that had significant impacts on our first model. Extracted factors were participant group (i.e., hearing ability), emotion expression level, interaction between participant group and emotion expression level, interaction between participant group and type of text response (i.e., “yes” or “no”), interaction between hearing ability, type of text response, and emotion expression level. The results confirmed what we found in the ANOVAs.

Overall, the ratings were more positive in hearing participants than in deaf participants [odds ratio = 2.54, 95% CI (1.66, 3.89), Wald χ^2^(1) = 18.3, *p* < 0.001]. The higher ratings of positivity were associated with more positive emotion expression with an odds ratio of 1.94 [95% CI (1.80, 2.10), Wald χ^2^(1) = 280.9, *p* < 0.001]. The effect was smaller in hearing participants than in deaf participants with an odds ratio of 0.73 [95% CI (0.67, 0.81)], Wald χ^2^(1) = 40.4, *p* < 0.001.

Hearing participants perceived the positive text response (i.e., “yes”) as more positive than they perceived negative (i.e., “no”) as negative with an odds ratio of 0.21 [95% CI (0.14, 0.31), Wald χ^2^(1) = 63.1, *p* < 0.001]. In contrast, deaf participants did not show such an asymmetry [odds ratio = 0.75, 95% CI (0.47, 1.20), Wald χ^2^(1) = 1.45, *p* = 0.23].

Furthermore, significant interactions between response type and emotion expression level were found both in hearing and deaf participants. In both groups, the increase of ratings with increasing emotion expression level was steeper for positive than for negative text response [odds ratio = 1.75, 95% CI (1.60, 1.90), Wald χ^2^(1) = 158.1, *p* < 0.001 for hearing participants; odds ratio = 1.15, 95% CI (1.03, 1.27), Wald χ^2^(1) = 6.41, *p* < 0.05 for deaf participants].

## Discussion

The present results showed that there was no significant effect related to participant hearing status in the judgment of facial expression, suggesting that the way hearing and deaf participants interpreted expression of emotion on faces did not differ. Past research has also suggested no difference between deaf and hearing participants in interpreting the emotional valence of facial expression using human facial pictures depicting various emotions (Watanabe et al., 2011). Our findings are consistent with these results and extended the understanding to non-realistic human faces (i.e., schematic faces and CG faces).

The findings from the present study also indicate that in terms of inferring real meaning of the verbal response, the emotion expressed on the face might qualify the meaning of what is explicitly stated as a verbal response to the question. For example, when the verbal response was “yes,” the real meaning was rated at approximately two and thus interpreted as “no” for the faces expressing the highest levels of negative emotion. Facial expressions serve as strong non-verbal cues in recognizing others’ intention (Ekman and Friesen, 1974; Friedman, 1979). The significant interactions between response type and facial emotion expression in the ANOVA and the ordinal regression model indicate an asymmetry in the contribution of facial expression depending on the response. In other words, we rely on facial expression in interpreting the text messages more when interpreting a positive than a negative response. This may indicate that we spontaneously assume that others may hide their real feeling in order to behave kindly or politely to us (politeness assumption), and thus in such a situation, we may tend to integrate non-verbal cues other than their direct response presented verbally. When others respond negatively, we tend to interpret their responses as their real meaning and thus make less use of non-verbal cues such as facial expression, as there is no reason for others to pretend to be unsociable.

As for the commonality and difference between deaf and hearing participants, the current results showed that (1) there was no difference in interpreting emotional valence from faces, (2) both groups were influenced by the facial expressions to infer the real meaning behind the text response, (3) the influence of facial expression was smaller when interpreting the text response that was expressing negative contents to the questioner in hearing participants, and (4) there was no such difference in deaf participants. For the influence of face type and conversational context, the ordinal logistic regression analysis showed that (5) no influence of facial type or conversational context was found in both participant groups, while (6) the interactions between facial type, context, and response or between context, response, and expression level were suggested for deaf participants only. *Post hoc* analyses following ANOVAs suggested that the influence of response type was observed only in CG faces in deaf participants.

In our results, the most pronounced difference in communication style between deaf and hearing people was the effect of positive emotion expression on interpreting the negative responses. Hearing participants tended to interpret negative response as having negative meaning, irrespective of the positivity of emotion expression (i.e., “no means no”). However, deaf participants tended to be influenced more by positive facial expression when interpreting the negative responses.

One possible reason could be that deaf people consider non-verbal visual cues (including facial expression) as more useful sources for interpreting verbal messages because they usually have less access to auditory cues. Hearing people integrate face and voice information in understanding others in everyday situations (Campanella and Belin, 2007), while the degree of cross-modal influence between facial expression and voice depends on culture (Tanaka et al., 2010). In the current study, our experimental condition provided verbal information as written texts presented on the monitor and thus did not provide prosodic sound information that could be used to infer emotion. However, this did not prevent participants from imagining the prosody of each verbal stimulus. Hearing people may weigh visual information differently than deaf people because they usually have access to auditory cues (e.g., prosodic sounds). More specifically, visual facial expressions might be more useful sources for deaf people for understanding emotions. This in turn might explain the smaller asymmetry (i.e., the relatively larger effect of positive facial expression on the negative messages). However, there are other possibilities that might explain the current findings, such as difference in conceptualization of politeness and exploratory strategy (e.g., eye-movement). Further research will be required to clarify this issue.

Our results suggested that there was no significant difference between face types. This implies that even simple schematic faces can be as strong non-verbal cues for modifying interpretations of text messages as realistic CG faces, which is consistent with research on emoticons and avatars (Walther and D’Addario, 2001; Derks et al., 2008). However, our results also showed that the influence of facial expression on interpretation of the text message differed depending on hearing experience or ability of participants and that this difference was found in particular when the text response was expressing negative content to the questioner. These findings indicate the inhomogeneous effect of facial emotion information on text messages and its interaction with the communication strategy of the receiver. Therefore, caution should be exercised when emoticons or expressive avatars are used as non-verbal cues in human–computer interaction and human–human interaction via information systems. Although, in the current study, we focused on the difference between hearing and deaf people, our findings that the integration of emotion expression might rely on the presumption of politeness might be extended to possible differences between cultures. Perception or expectation of politeness and how it is conceptualized in the conversation might differ between cultures (Matsumoto, 1988; Haugh, 2004). In particular, as Matsumoto (1988) reported, the concept of “face” (in pragmatics) in Japanese culture may differ from that of other cultures, and this might represent a consideration for the present findings.

In conclusion, facial expressions influenced the interpretation of the response that was verbally presented as text. The influence of positive facial expressions on the perception of negative verbal response was smaller compared to that of negative facial expressions on the perception of positive verbal response. Although the perception of facial expression did not differ depending on hearing status, the influence of positive/negative emotion expressions on the perception of negative/positive verbal response was less asymmetrical in deaf participants compared to that in participants with normal hearing. This difference might be due to the difference in availability and usage of prosodic sound and facial expression (i.e., feature/holistic processing of faces in deaf/hearing participants) in inferring the real meanings from verbal messages. Although we focused on the effect of facial expression on interpretation of text messages in the current study, our results could also be interpreted in other ways, that is, text messages may affect the interpretation of the facial emotion expressions. These possibilities require further investigations.

### Conflict of Interest Statement

The authors declare that the research was conducted in the absence of any commercial or financial relationships that could be construed as a potential conflict of interest.
